# Erectile Dysfunction in Veterans with Post-Traumatic Stress Disorder

**DOI:** 10.1192/j.eurpsy.2025.1945

**Published:** 2025-08-26

**Authors:** Y. Ben Othmene, A. Baatout, W. Kabtni, K. Kefi, S. Eddif, H. Kefi, A. Oumaya

**Affiliations:** 1 Psychiatry, Military Hospital, Tunis, Tunisia

## Abstract

**Introduction:**

The mental health burden of post-traumatic stress disorder (PTSD) is significant for all those affected, with a higher incidence among veterans due to military trauma and the particular strains of military duty. It results in detrimental effects on life quality and functional impairment in various domains, including sexual dysfunction (SD).

One of the most prevalent yet underreported sexual dysfunction in Tunisian veterans is erectile dysfunction (ED).

**Objectives:**

The aim of the current study was to assess the prevalence of ED and its severity among Tunisian veterans with PTSD versus those without it.

**Methods:**

A cross-sectional descriptive and analytical survey was conducted between September and November 2024 on Tunisian veterans seeking consult, using a data file and 2 self-report questionnaires :

The PTSD Checklist for DSM-5 (PCL-5) to assess current PTSD symptoms with a cut-off score of 33 or higher to detect PTSD cases.

The IIEF-5 (International Index of Erectile Function 5) to evaluate ED with six categories: [1-4]: uninterpretable, [5-7]: severe, [8-11]: moderate, [12-16]: mild to moderate, [17-21]: mild and [22-25]: no ED.

To analyze the obtained data, IBM SPSS was used.

**Results:**

Fourty veterans were enrolled in this study with an average age of 38.5 [24-61] years. The majority (67.5%) were married, followed by 25% single individuals, 5% divorced and 2.5% in a relationship. Most of the participants (77.5%) were smokers and 25% reported alcohol consumption, with 20% of them being regular drinkers and 80% consuming alcohol occasionally. None reported using cannabis or other illicit drugs. Regarding medical history, 32.5% had health issues with the most common being varicocele, diabetes, arterial hypertension and myocardial infarction.

Among the veterans, 50% had PTSD. Ninety-five percent had received psychiatric follow-up, and 78.9% were on antidepressants. In the control group with no PTSD (50%), 85% had a psychiatric follow-up, with 76.5% taking antidepressants.

In patients with PTSD, 75% reported ED, while 15% had no SD and 10% had an uninterpretable score. ED was mild to moderate in 46.7%, mild in 33.3% and moderate in 20%. No severe cases of ED were observed in this group.

In contrast, in patients with no PTSD, ED was only reported in 60% of cases and was even severe in 33.3% of patients.

No significant correlation was found between ED and PTSD (p= 0.3).

The prevalence of ED in the overall sample was 67.5% with only 11.1% of them using sexual enhancers.

**Conclusions:**

This study suggests that while PTSD may exacerbate ED in some individuals, other factors such as comorbid psychiatric or medical condition and the use of a variety of medications may play a more significant role in the underlying cause. Given its significant impact on quality of life, early screening and treatment are essential.

Additionally, further research is needed to understand the underlying causes of ED and to develop more targeted interventions.

**Disclosure of Interest:**

None Declared

